# Inhibition of VEGF-C Modulates Distal Lymphatic Remodeling and Secondary Metastasis

**DOI:** 10.1371/journal.pone.0068755

**Published:** 2013-07-16

**Authors:** Alvin Gogineni, Maresa Caunt, Ailey Crow, Chingwei V. Lee, Germaine Fuh, Nicholas van Bruggen, Weilan Ye, Robby M. Weimer

**Affiliations:** 1 Department of Biomedical Imaging, Genentech Inc., South San Francisco, California, United States of America; 2 Department of Molecular Biology, Genentech Inc., South San Francisco, California, United States of America; 3 Department of Antibody Engineering, Genentech Inc., South San Francisco, California, United States of America; Istituto Superiore di Sanità, Italy

## Abstract

Tumor-associated lymphatics are postulated to provide a transit route for disseminating metastatic cells. This notion is supported by preclinical findings that inhibition of pro-lymphangiogenic signaling during tumor development reduces cell spread to sentinel lymph nodes (SLNs). However, it is unclear how lymphatics downstream of SLNs contribute to metastatic spread into distal organs, or if modulating distal lymph transport impacts disease progression. Utilizing murine models of metastasis, longitudinal *in vivo* imaging of lymph transport, and function blocking antibodies against two VEGF family members, we provide evidence that distal lymphatics undergo disease course-dependent up-regulation of lymph transport coincidental with structural remodeling. Inhibition of VEGF-C activity with antibodies against VEGF-C or NRP2 prevented these disease-associated changes. Furthermore, utilizing a novel model of adjuvant treatment, we demonstrate that antagonism of VEGF-C or NRP2 decreases post SLN metastasis. These data support a potential therapeutic strategy for inhibiting distant metastatic dissemination *via* targeting tumor-associated lymphatic remodeling.

## Introduction

Metastatic disease accounts for the majority of deaths associated with solid tumors [1], [2]. Even within patient populations where primary lesions are fully resected and adjuvant chemotherapy or local radiotherapy administered, metastatic spread and mortality is not prevented [3]–[9]. The disconnect between therapy and cure highlights a long-standing challenge in cancer treatment: the likely escape of pro-metastatic cells from primary tumors prior to clinical presentation of disease. Thus, therapeutic approaches that target cell dissemination may provide clinically tractable means to mitigate cancer progression.

The route of metastatic cancer cell dissemination is context-specific, however several lines of clinical data highlight a central role for tumor-associated lymphatics in metastatic disease within a range of cancers. The lymph system is an organized hierarchal network of blunt-ended lymphatic capillaries, precollector vessels, and collecting vessels that drain lymph and transport immune cells to lymph nodes. Fluid absorption occurs within capillaries; while precollector and collecting vessels are associated with smooth muscle cells, which contribute contractility, and bi-leaflet valves that controls unidirectional propulsion of lymph through this network [1], [2], [10]. In metastatic breast, prostate, colon and head and neck squamous cell carcinoma patient populations, tumor-associated (peri- and intra-tumoral) lymphatics exhibit features of remodeling: increased lymphatic endothelial cell proliferation, vessel density and dilation [3]–[12]. Within these patient populations tumor cells can be detected in associated lymphatics along with metastatic lesions within draining SLNs–the latter provides direct evidence of metastatic cell transit through lymphatics. Furthermore, there is a high degree of correlation between SLN and distant organ metastases, while primary lymphatic vessel density correlates with metastasis frequency and clinical outcome [13]–[18].

The notion of tumor to lymphatic signaling in metastatic disease is also supported by clinical findings that in metastatic breast, prostate, colon and head and neck squamous cell carcinoma, expression of the pro-lymphangiogenic growth factors VEGF-C or -D in tumor and associated stromal cells correlates with increased incident of metastatic disease [11], [19]. VEGF-C and -D are members of the vascular endothelial growth factor family and promote lymphaniogenesis *via* binding and activating their cognate tyrosine kinase receptors VEGFR2 and 3 [20]. Full VEGF-C activity also requires its co-receptor NRP2 [21]. Perhaps, expression of these pro-lymphangiogenic molecules promotes lymphatic involvement, which facilitates cell dissemination.

Experimental data in preclinical murine models of metastatic disease also suggest a key role of lymphatics in tumor cell dissemination [22]. Xenograft tumors generated from cell lines expressing VEGF-C or -D, for example, promote tumor-associated lymphangiogenesis and exhibit metastatic spread to SLNs and distant organs [23]–[25]. Peritumoral lymphatics in these models exhibit structural alterations reminiscent of clinical pathology, specifically increased vessel dilation [26]–[28]. This structural change could account for the reported increased lymph transport in primary tumor-associated lymphatic [27], [29]–[31]. Furthermore, inhibition of pro-lymphangiogenic factors, such as VEGF-C, VEGF-D, NRP2 and VEGFR3, prior to disease spread can reduce the occurrence of metastatic lesions within SLNs [20], [28], [32]–[35], suggesting that antagonism of this pathway may provide an approach to mitigate metastatic disease. However, as tumor cells traverse through lymphatic vessels downstream of SLNs to reach distant sites or systemic circulation, it remains unknown if lymphatics distal to SLNs undergo disease-related remodeling, or how remodeling contributes, if at all, to metastatic disease spread. Furthermore, it remains untested whether inhibition of pro-lymphangiogenic signaling after tumor cell seeding in SLNs could prevent further spread to target organs.

To address these questions we utilized a longitudinal *in vivo* imaging strategy to assess physiological changes in post-SLN lymphatic vessels in the context of metastatic disease. Our study built upon the collective knowledge that pro-lymphangiogenic signaling modulates structure and function of primary and regional tumor-associated lymphatic vasculatures [31], [36], [37]. We expand upon this knowledge by demonstrating that tumor-associated distal lymphatics downstream of SLNs undergo functional and structural remodeling during disease progression, which can be mitigated *via* treatment with function blocking antibodies to VEGF-C or NRP2, but not VEGF-A. In addition, we demonstrate, utilizing a novel model of adjuvant therapy, that VEGF-C and NRP2 antagonism reduces metastatic spread beyond SLNs. Together, our results provide the potential basis for a clinical approach in the adjuvant treatment of cancer.

## Materials and Methods

### Animals

All studies were approved and conducted in accordance with institutional guidelines for the care and use of laboratory animals governed by Genentech’s Institutional Animal Care and Use Committee (IACUC). BALB/c nude females 6 to 8 week of age were purchased from Charles River Laboratories (Hollister, CA).

### *In vivo* Imaging of Lymph Transport

Animals were anesthetized with isoflurane (1.5%, 1 L/min flow rate), immobilized on a heated platform to maintain body temperature at 37°C and injected intradermal with a 15 µl bolus of 5 mg/mL Alexa680-70 KDa dextran (Molecular Probes) in PBS at the base of the tail, 5 mm lateral to the rectum. When visualized by whole animal near-infrared fluorescence imaging (Kodak 400 FX Pro, 630 nm excitation, 700 nm emission, 1 sec exposure, 2× binning), fluorescent signal is detected at the injection site and within lymph vessels associated with the inguinal to axillary drainage path. To detect pulsatile movement of lymph through the inguinal to axillary vessel animals were mounted under a 4× objective lens (Olympus) of an epifluorescence microscope (Prairie Technologies) equipped with a Cy5.5 filter set (Chroma) and CCD camera (Olympus, model S97827) controlled by Micromanager (N.I.H.). Images (348×260 pixels, 4.31 µm per pixel) were acquired at 4 Hz for 5 minutes. Prior to the start of image acquisition, animal respiratory and heart rates were monitored using a pulseoximeter (MouseOx system, Starr Life Sciences Corp), and anesthesia adjusted to ensure similar physiological conditions between animals and across experiments. Pulsatile movement of lymph was detected by quantifying the average pixel intensity through time within a region of interest over the lymph vessel using TimeSeriesAnalyzer in ImageJ (N.I.H.). A positive peak in intensity was defined as one pulsatile event; frequency of pulsations per animal was determined by dividing the number of detected events by the duration of imaging (typically 5 minutes). For display in the figures, fluorescence intensity traces over time were normalized within a time series as follow: Normalized fluorescence intensity for a given time point x = (F_tx_−F_min_)/(F_max_−F_min_).

### Xenograft Metastatic Tumor Models

To study lymph function in the context of metastatic disease we utilized the C6 rat gliobastoma and 66c14 breast carcinoma cell lines (ATCC), which were previously shown to express VEGF-C and undergo metastatic spread in murine models [35], [38], [39]. For implantation, cells were grown in culture (DMEM, 10% FBS, 37°C, 5% CO_2_ 95%), harvested following trypsinization and resuspended in PBS (1×10^5^ cells per µl). For implantation at indicated locations within BALB/c females, 5 µL of cell suspension was injected subcutaneously. Animals exhibiting palpable sentinel lymph node tumors one-week post implantation (i.e. before the first imaging session) were excluded, as this likely represented direct injection of cells into the draining lymphatics. 66C14 xenograft tumors exhibited slower growth than C6 xenograft tumors (data not shown), therefore imaging experiments utilizing this cell line were conducted at 5 weeks post implantation when tumor reached an average size comparable to C6 xenograft tumors at week 3.

Adjuvant disease was modeled by implanting the xenograft tumor into the interdermal space at the tip of the ear (1×10^5^ cells in 1 µl of PBS), which enables full resection of the primary lesion. At day 12 post-implantation, primary tumors were surgically excised and animals randomized, 20 mice per treatment cohort and dosing initiated with once a week frequency until end of study. At day 26, 14 days after treatment initiation, animals were sacrificed and lymph nodes and lungs collected for analysis. Primary metastatic lesions were measured by weighing the sentinel lymph node; lung metastasis was quantified based on visual detection of lung lesions. To ensure that implantation of cells did not directly introduce cells into hematogenous or lymphatic circulation due to increased extra-vascular fluid pressure, a separate cohort of animals were implanted with cells as described above then 24 hours post-implantation the primary tumor was excised. These mice exhibited no signs of metastasis in the SNL or lung 4 weeks post-resection (data not shown).

### Antibody Treatment Conditions

For all antibody treatment studies animals were dosed with 10 mg/kg isotype control anti-ragweed antibody (Genentech, lot #1428), 40 mg/kg anti-NRP2^B^, 10 mg/kg anti-VEGF-A (clone B20-4.1.1) or 10 mg/kg anti-VEGF-C (clone VC1.12 or VC4.5) by intraperitoneal injection as indicated. Anti-NRP2^B^ was dosed at higher concentration, compared to anti-VEGF-A and anti-VEGF-C, due to differences in pharmacokinetic properties between the antibodies (data not shown).

### Morphometric Quantification of Lymph Vasculature

For all morphometric analysis, image acquisition and analysis was carried out blinded with regard to experimental conditions. Lymphatic valves were labeled *in vivo* by 25 µl of 1 mg/mL intradermal injection of FITC-tomato lectin (Vector Laboratories) at the base of the tail. Inguinal to axillary lymph vessels were collected and imaged whole mount by epifluorescence stereoscope (Leica) equipped with a GFP filter set (Chroma) and CCD camera (Leica, DFC360FX) at room temperature. Valve density was estimated by counting the number of stained valves per mm of vessel within the field of view imaged (696×520 pixel, 2.72 µm/pixel).

To visualize lymphatic vessel associated smooth muscle cells, the lymphatic vessels draining from the inguinal to the axillary lymph node were dissected whole mount, fixed, immunostained using anti-alpha smooth muscle cell actin (αSMA), then imaged by epifluorescence microscopy (Microscope, Axioplan2, Carl Zeiss, Inc.; CCD, HQ2 Photometrics; software, SlideBook v. 5.0) at room temperature. Specifically, following dissection tissues were washed in PBS 3 times for 20 minutes and blocked overnight at 4°C in blocking buffer (PBS, 2% chicken serum, 2% donkey serum 0.5% Triton X-100, 1% BSA, 0.1% cold fish skin gelatin, 0.05% tween-20, and 0.05% sodium azide), incubated overnight at 4°C with 1∶500 anti-alpha smooth muscle actin-cy3 (Sigma) in blocking buffer, then washed in PBS and 0.3% Triton X-100 3 times, counterstained with DAPI, and mounted with fluoromount G (EMS Sciences). For quantification of vessel width, 3 inter-valve regions per vessel were imaged per mouse using a 5× objective lens (696×520 pixel, 2.65 µm/pixel). To quantify the density of αSMA-positive cells, 2 inter-valve regions per mouse were imaged using a 40× objective (696×520 pixel, 0.65 µm/pixel). The density of SMA-positive cells per vessel length was estimated by quantifying the number of SMA DAPI co-stained cells along the top focal plane of the vessel.

### Statistical Analysis

Statistical analysis was performed using JMP version 9.0.2 (SAS). For statistical comparison of data sets with two groups, Student’s t-tests were performed; for multi-group analysis, significance was assessed using one-way ANOVA with *posthoc* Tukey-Kramer HSD test. Within figures, * represents *p*<0.05; ** represents *p*<0.001. In all cases where significance was assessed, the experiments were repeated to confirm the result (data not shown).

## Results

### Lymph Transport Increases with Metastatic Disease Course in Distal Tumor-associated Lymphatics

**Figure 1:**
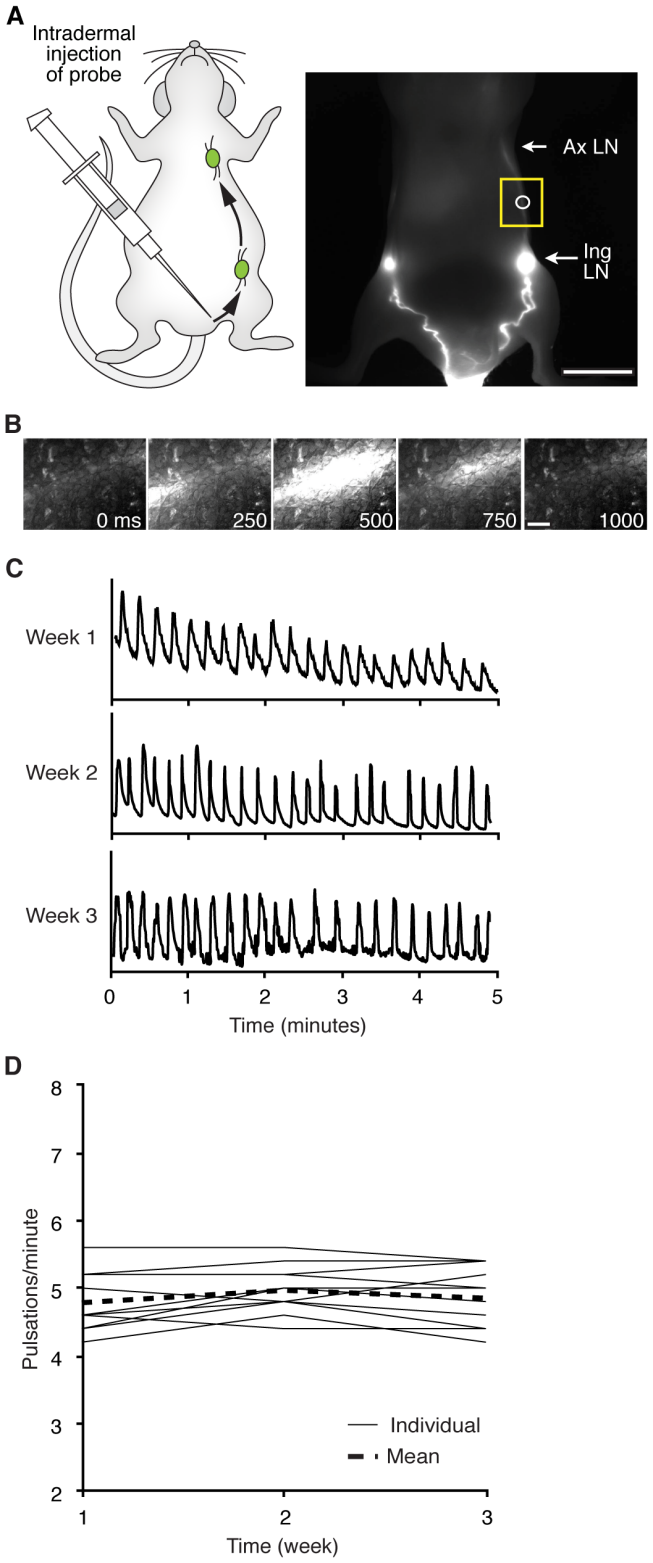
Longitudinal measurement of lymph pulsatile movement. (**A**) Schematic (left) and near-infrared fluorescence image (right) showing injection sites of fluorophore and lymphatic drainage pathway to Inguinal (Ing) and Axillary (Ax) lymph nodes (LNs). (□) denotes the region imaged to capture dynamic lymph transport. (**○**) represents the region of interest (ROI) where fluorescence intensity is quantified to detect lymph pulsatile movement. (**B**) Time series illustrating a bolus of probe-laden lymph being propelled through the lymphatic vessel. (**C**) Normalized fluorescence intensity plotted as a function of time within a ROI measured on three consecutive weeks. Pulsatile events are detected as peaks in the trace. (**D**) Pulsatile frequency per animal and as the mean across animals per week for three consecutive weeks: week 1, 4.78±0.13; week 2, 4.97±0.10; week 3, 4.85±0.13 pulsations/minute ± SEM, n = 12. Scale bar equals 1 cm in **A** and 1 mm in **B**.

Lymph is transported in a unidirectional manner through collecting lymphatic vessels *via* pulsatile movement [21], [40], [41]. To quantitatively assess the pulsatile movement of lymph in murine tumor models, we utilized near-infrared fluorescence imaging to visualize the transport of a high molecular weight fluorescent probe conjugate (Alexa680-70 kDa Dextran). Intradermal injection of probe at the base of the tail resulted in stereotypical uptake *via* lymphatic capillaries, followed by transport and clearance through the draining inguinal to axillary lymphatic network [22], [42] (Figure 1A). The pulsatile movement of lymph through collecting vessels was visualized by high-speed sampling (4 Hz), and appeared as boluses of fluorophore passing through a vessel (Figure 1B). Consequently, detection of fluorescence intensity spikes at a given vessel location (Figure 1C), such as the lymph vessel connecting the inguinal to axillary lymph node (LN), provided a reproducible method to quantify the rate of pulsatile movement longitudinally (Figure 1D). Importantly, this method enabled detection of physiological changes in lymph transport across a range of conditions (Figure S1). However, this approach does not enable reproducible assessment of the trace waveform, likely due to the inability to consistently identify the same relative position within a lymphangion when imaging through the dermis.

**Figure 2:**
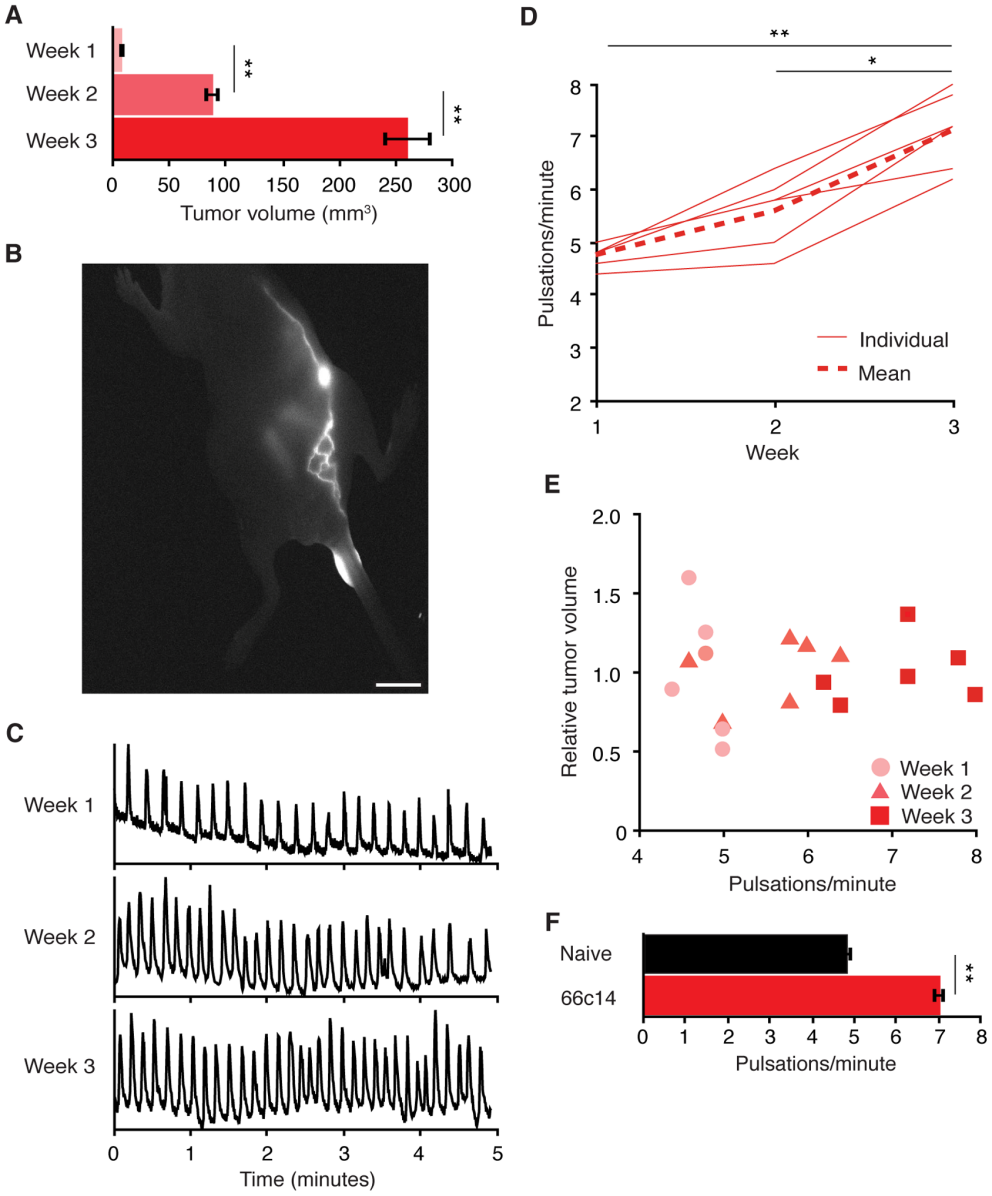
Lymph pulsatile movement increases with metastatic disease progression. (A) Average volume of C6 tail xenograft tumors at indicated times post-implantation. Bar graph represents mean ± SEM, n = 6 animals. (B) Near-infrared fluorescence image following intratumoral injection of probe confirming tumor lymph drainage through the inguinal lymph node (Ing LN). Scale bar equals 1 cm. (C) Representative traces of normalized fluorescence intensity measured from labeled Ing to Ax lymph vessels in tumor-bearing animals at indicated weeks post-implantation. (D) Pulsatile frequency per animal and as the mean across animals per week after tumor implantation: week 1, 4.77±0.09; week 2, 5.60±0.27; week 3, 7.13±0.30 pulsations/minute ± SEM, n = 6 animals. (E) Relative individual tumor volume per week plotted as a function of pulsatile frequency. Relative tumor volumes were calculated by dividing individual tumor volumes by the mean tumor volume for the corresponding week. (F) Pulsatile frequency in naïve versus 66c14 tail xenograft tumor bearing animals five weeks post implantation: 4.84±0.17 versus 7.04±0.17 pulsations/minute, n = 5 animals per group, bar graph represents mean ± SEM.

We applied this imaging strategy to study the relationship between metastatic disease progression and lymph transport in tumor-associated lymphatics downstream of SLNs, also referred to as distal lymphatics. Tumor metastasis was modeled using the rat C6 glioblastoma cell line to generate xenograft tumors implanted at the base of the animals tail. C6 cells exhibit a propensity to metastasize to distant organs, including the lung [23], [24], [35], and are commonly used to study metastatic processes. Implantation of 5.0×10^5^ cells subcutaneously at the base of the tail generated tumors that exhibited logarithmic growth (Figure 2A) and induced metastases within lungs by three weeks post implantation (data not shown). Intratumoral injection of fluorescent tracer confirmed that lymph in the primary tumors drain into the inguinal to axillary lymph network (Figure 2B). Longitudinal assessment of lymph transport over the course of tumor growth and metastasis revealed a time-dependent increase in the rate of lymph pulsatile movement in tumor-bearing mice (Figure 2, C and D). Specifically, pulsatile frequency increased ∼17% between week 1 and 2, and ∼27% between week 2 and 3 post implantation. At a given time point, pulsatile frequency was not related to tumor size (Figure 2E), suggesting that increased pulsatile lymph movement reflects time-dependent changes during disease progression other than increased tumor volume. Furthermore, animals bearing xenograft tumors derived from the 66c14 mouse metastatic breast cancer cell line also exhibited an increase in pulsatile frequency (Figure 2F), suggesting that modulation of pulsatile movement may represent a common pathophysiology in metastatic disease.

**Figure 3:**
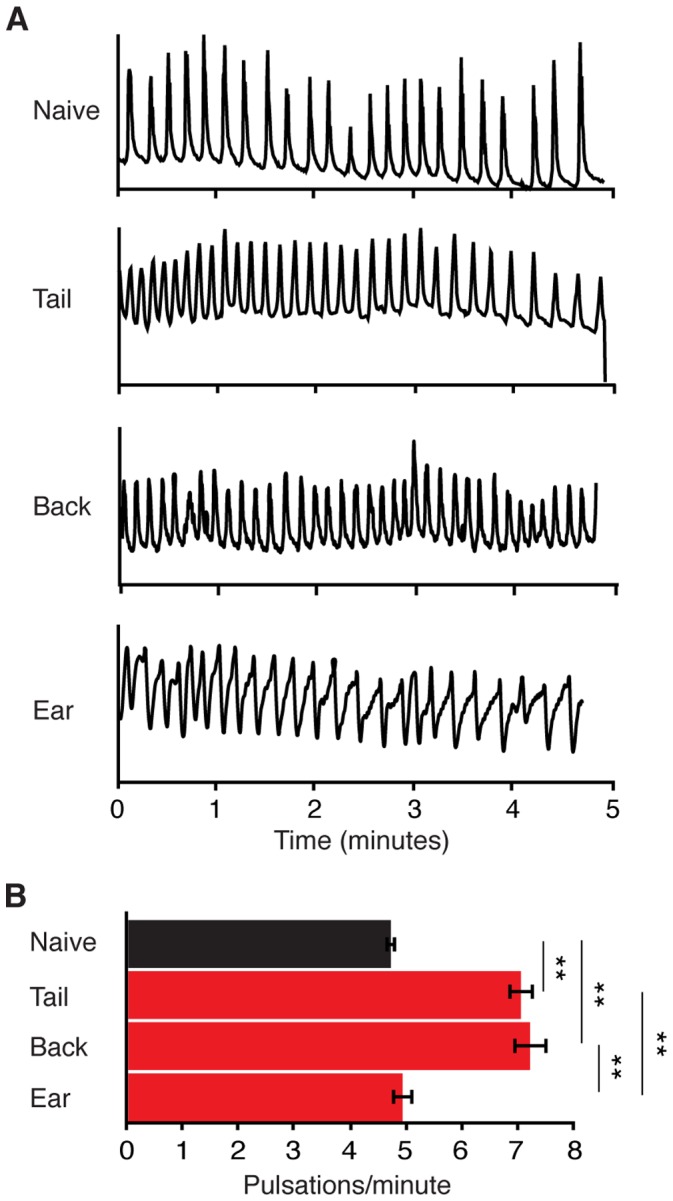
Tumor-induced lymphatic modulation is specific to tumor-associated lymphatics. (A) Representative traces of normalized fluorescence intensity measured from labeled inguinal to axillary lymph vessels in naïve and C6 tumor-bearing animals three weeks post xenograph tumor implantation at indicated locations. (B) Average pulsation frequency in naïve versus tumor location: naïve; 4.75±0.17; tail, 7.08±0.24; back, 7.24±0.31; ear 4.96±0.31 pulsations/minute. Bar graphs represent mean ± SEM, n = 4 and 5 animals per group for naïve and tumor-bearing groups, respectively.

To further test the hypothesis that up-regulation of lymph transport in the distal lymphatics represents disease-related pathophysiology, we implanted C6 xenograft tumors at various locations within animals then measured pulsatile frequency within the inguinal to axillary vessel. Xenografts implanted on the back flank, which drain lymph through the inguinal to axillary network and metastasize to the lung, resulted in an increase in pulsatile frequency to a similar extent as tail-implanted tumors (Figure 3). However, xenografts implanted into the ear, which also metastasize to the lung but drain lymph through the cervical to axillary network, did not result in up-regulation of pulsatile frequency within the inguinal to axillary vessel. These data suggest that lymph transport is increased as a function of disease progression in distal tumor-associated lymphatic vessels–a pathophysiological response that could aid in metastatic cell dissemination.

### VEGF Family Members Regulate Lymph Transport in Distal Tumor-associated Lymphatics

**Figure 4:**
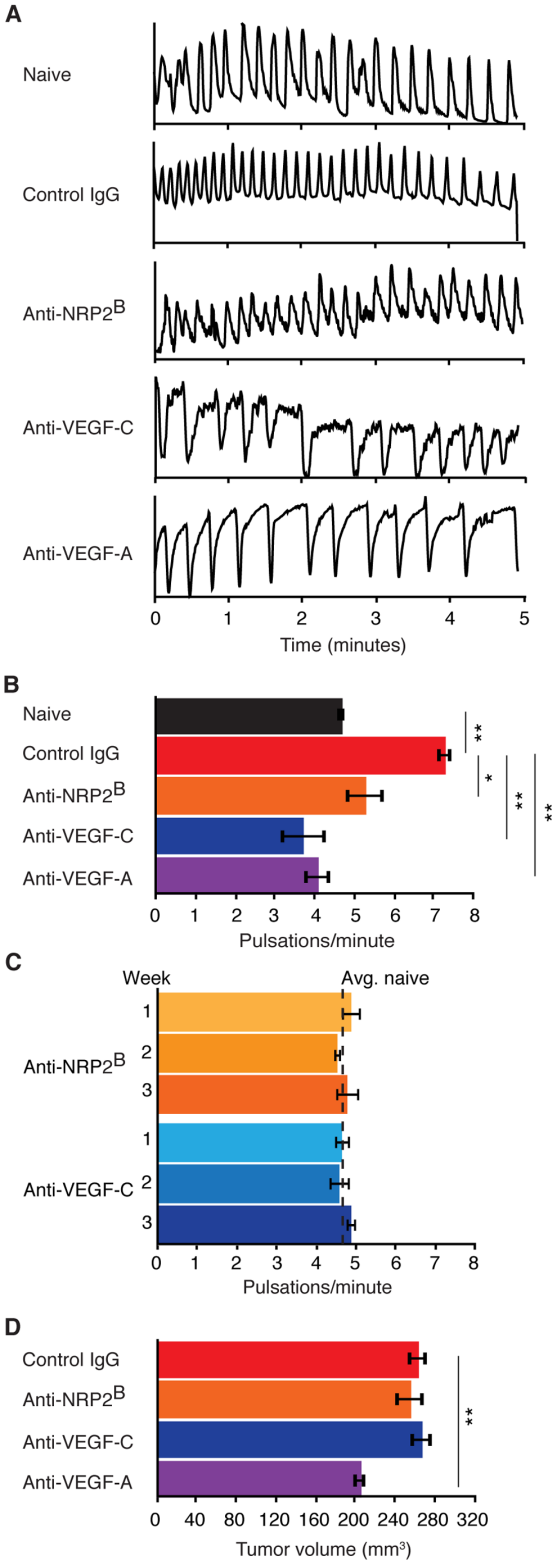
Inhibition of VEGF family members prevent up-regulation of pulsatile lymph movement. (A) Representative normalized fluorescence intensity traces collected from the Ing to Ax lymph vessel in naïve or C6 tail xenograft tumor-bearing animals three weeks post tumor implantation. Treated animals were dosed weekly with control or function blocking antibodies against NRP2, VEGF-C or VEGF-A starting two days after xenograft implantation. (B) Pulsatile frequency per tumor and treatment condition: naïve, 4.68±0.10; control IgG, 7.28±0.19; anti-NRP2^B^, 5.28±0.49; anti-VEGF-C (VC1.12), 3.72±0.57; anti-VEGF-A, 4.08±0.34 pulsations/minute, n = 5 per group. (C) Average pulsatile frequency in naïve animals treated with anti-NRP2^B^ or anti-VEGF-C (VC4.5), n = 4 and 6 animals, respectively. Animals were dosed and imaged weekly for three weeks with the first imaging session starting five days after the first dose. Dashed line represents the average pulsatile frequency, 4.64 pulsations/minute, observed in naïve animals across multiple experiments (n = 30 animals, 6 experiments). (D) Average tail xenograft tumor volume three weeks after implantation in animals dosed once a week for three weeks with indicated antibody: control IgG, 263.30±9.90; anti-NRP2^B^, 255.59±14.89; anti-VEGF-C (VC1.12), 267.78±11.75; anti-VEGF-A, 205.49±7.01 mm^3^, n = 5 per group. Bar graphs represent mean ± SEM.

While little is known regarding the signaling pathways regulating the pulsatile movement of lymph, recent work has begun to elucidate the role of VEGF family members in regulating lymphatic development. During development, VEGF-C binds the tyrosine kinase receptor VEGFR3 and its co-receptor NRP2 to promote lymphatic endothelial cell proliferation and vessel sprouting–similar to the canonical VEGF-A-VEGFR2/NRP1 pathway that regulates blood vascular development [26]–[28], [43], [44]. To test if VEGF family members contribute to the up-regulation of lymph transport associated with disease progression, we dosed C6 tail xenograft tumor-bearing animals with function blocking antibodies to NRP2 [35], called anti-NRP2^B^ herein, VEGF-C (clone VC4.5 [Caunt et al, in preparation] or VC1.12, Figure S2), or VEGF-A [45] weekly beginning two days after xenograft implant. Both anti-VEGF-C antibodies inhibit binding of VEGF-C to VEGFR3 and inhibit VEGF-C induced cell migration and proliferation *in vitro* to comparable levels, therefore were used interchangeably throughout this study. In contrast to IgG control, chronic treatment with anti-NRP2^B^, anti-VEGF-C (VC1.12) or anti-VEGF-A antibodies significantly reduced pulsatile frequency in tumor-bearing animals (Figure 4), with anti-VEGF-C and -A exhibiting greater effect than anti-NRP2^B^. Similar results were also observed with chronic treatment of anti-VEGF-C clone VC4.5 (data not shown). The different magnitude of effect between anti-VEGF-C and anti-NRP2^B^ could reflect the incomplete dependency of VEGF-C signaling on NRP2.

The reduction of distal lymph movement with VEGF axis inhibition does not reflect general systemic down regulation of lymphatic pulsation, as chronic treatment of naïve animals with anti-NRP2^B^ or anti-VEGF-C did not result in a decrease in pulsatile frequency (Figure 4C). Nor does the inhibition of lymph movement relate to inhibition of tumor growth as anti-NRP2^B^ or anti-VEGF-C did not significantly reduce tumor volume (Figure 4D). Rather, these results are consistent with the hypothesis that signaling of VEGF family members is required for disease-related modulation of lymph transport.

### VEGF-A Inhibition Normalizes Lymph Transport Secondarily and as a Consequence of Modulating Tumor Vasculature

**Figure 5:**
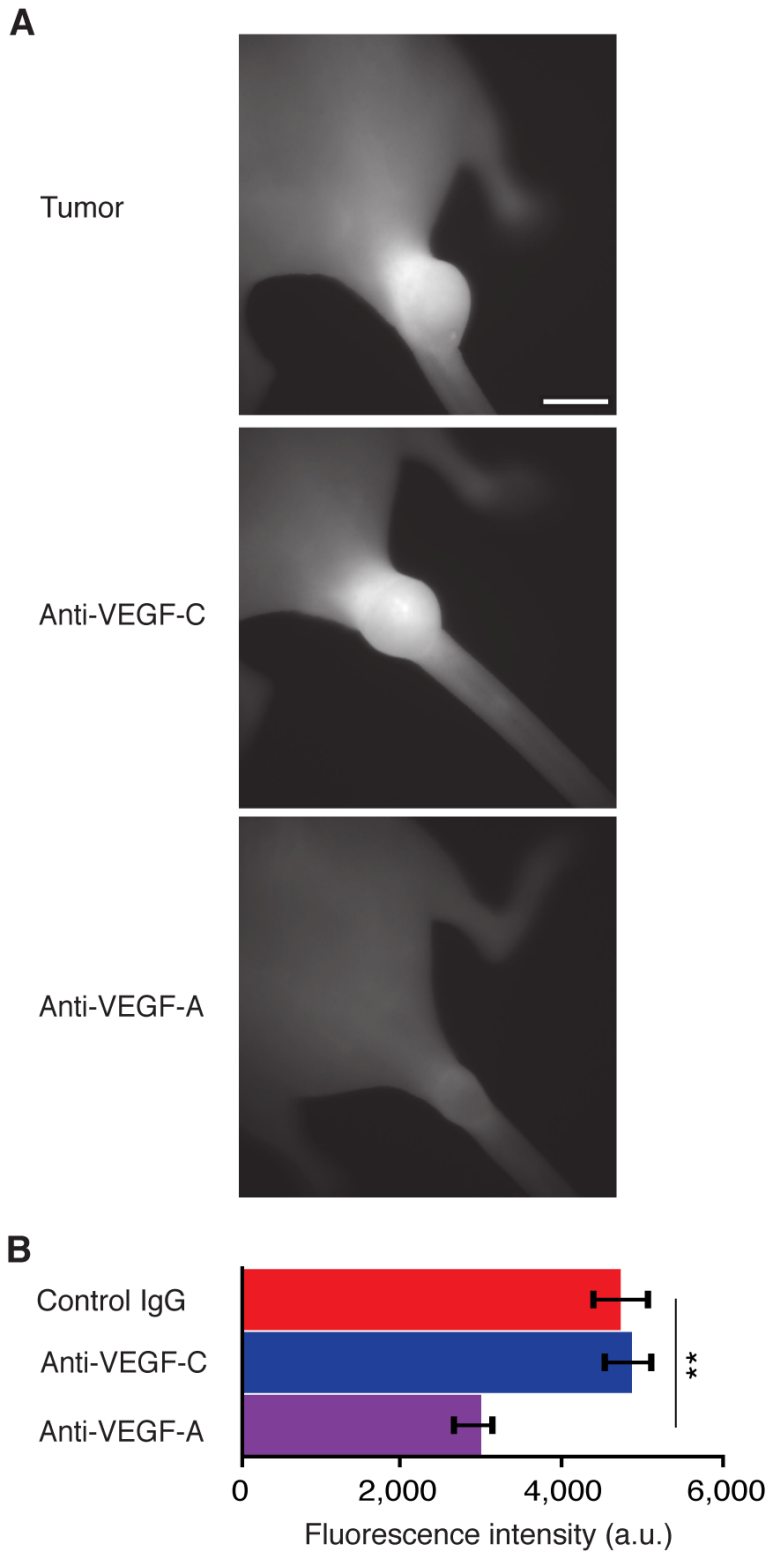
Tumor vascular permeability is reduced in anti-VEGF-A but not anti-VEGF-C treated animals. (A) Representative near-infrared fluorescence images of C6 tail xenograft tumors from indicated groups imaged 24 hours post 100 µL I.V. injection of AngioSense680. Animals were dosed weekly with indicated antibody starting two days post xenograft implantation, then imaged three weeks post implantation. Scale bar equals 5 mm. (B) Average fluorescence intensities within xenograft tumors across treatment conditions: control IgG, 4,746.75±388.66; anti-VEGF-C (VC4.5), 4,888.58±282.69; Anti-VEGF-A, 3,005.78±193.09. Bar graphs represent mean ± SEM, n = 6 animals per group.

Given the known effect of VEGF-A in tumor vascular biology, the normalization of lymph pulsatile movement through inhibition of VEGF-A raises the possibility that altering vascular physiology within the primary tumor may modulate distal lymph transport as a secondary consequence. To test this hypothesis we assessed vascular permeability within C6 tail xenograft tumors across treatment conditions. Specifically, the large molecular weight fluorescence blood pool probe AngioSense680 was administered I.V., allowed to circulate and clear for 24 hours then its relative abundance quantified within tumors *via* whole animal near-infrared fluorescence imaging. Due to the differential between systemic and interstitial clearance rates, fluorescent blood pool probes will transiently accumulate within tissues, which corresponds to the permeability of the target tissue vascular endothelium [46]. In anti-VEGF-A treated animals, the average fluorescence intensity within primary tumors was roughly 50% lower than that in control IgG treated animals (Figure 5). This apparent reduction in extravasated probe is consistent with the previously characterized role of VEGF-A in regulating vascular permeability [47], [48]. In anti-VEGF-C treated animals, however, the average fluorescence intensity within primary tumors did not differ from IgG control. These data taken together suggest that 1) the normalization of lymphatic transport in anti-VEGF-A treated animals likely reflects alterations in tumor vascular physiology: reduced vascular leak resulted in less interstitial fluid to transport; and 2) VEGF-C and NRP2 regulate lymph transport in distal tumor-associated lymphatic vessels through different mechanism than VEGF-A.

### Anti-VEGF-C-induced Changes in Distal Tumor-associated Lymphatic Function is not a Consequence of Lymphatic Density Changes in Primary Tumors

Since the VEGF-C pathway plays an important role in regulating lymphangiogenesis in primary tumors [23], [27], [32]–[35], we asked if the functional impact of VEGF-C inhibition on the lymphatics distal to the SLN was secondary to structural changes in primary tumors. Interestingly, we observed extremely low number of LYVE1-postivie intratumoral lymphatics when C6 tumors were implanted in the tails (Figure S3A and B). Quantification of peritumoral lymphatic vessel density did not reveal a significant alteration in primary tumor-associated lymphatic vessels with anti-NRP2^B^ or anti-VEGF-C treatment (Figure S3C). These findings are different from C6 tumors implanted in the flank [35], indicating that the micro-environment has a major impact on intratumoral lymphangiogenesis. We also measured peritumoral lymphatic vessel diameters and found no treatment effect (Figure S3D). Our analysis of primary tumors suggests that the functional impact of anti-VEGF-C on lymphatic vessels distal to SLNs is not a consequence of modulating lymphatic vessel density immediately associated with primary tumors. However, this does not rule out the possibility that VEGF-C inhibition alters lymph transport within intra- or peri-tumoral lymphatics.

**Figure 6:**
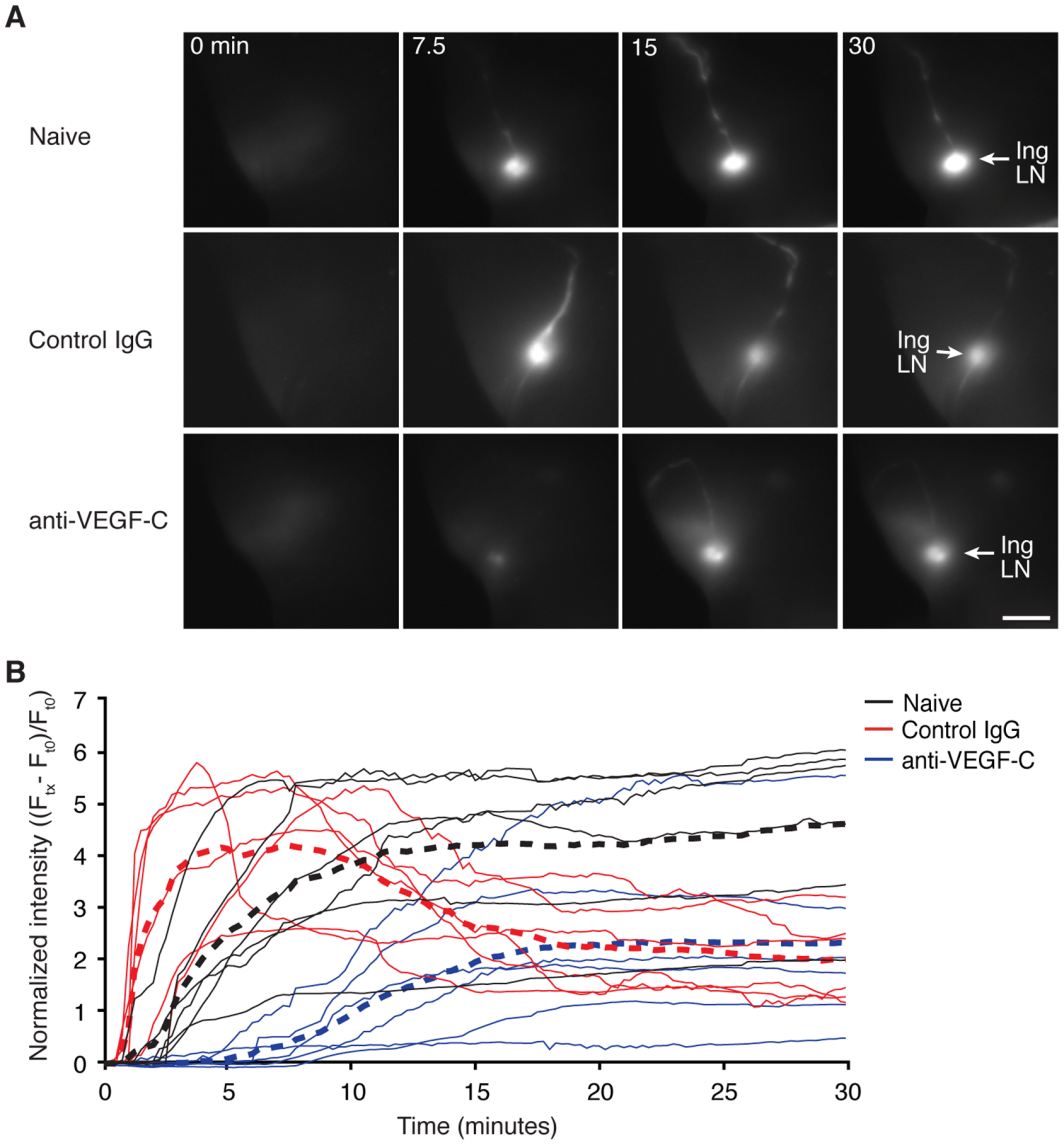
Lymph transport is increased distal to primary tumors and prevented with antagonism VEGF-C. (A) Representative near-infrared fluorescence images of inguinal lymph nodes (Ing LN) at indicated times post initiation of constant (5 µL/min for 15 minutes) intradermal infusion of fluorescent probe. Naïve or C6 tail xenograft tumor-bearing animals were dosed weekly for three weeks with either control IgG or anti-VEGF-C (VC4.5) antibodies starting two days after xenograft implantation. Scale bar equals 5 mm. (B) Quantification of fluorescence intensities within a region of interest containing the inguinal LN reveals that the average time to peak in fluorescence intensity in tumor-bearing animals is roughly half of that required in naïve animals. Anti-VEGF-C dosing reduced the rate of probe accumulation in tumor-bearing animals. Thin lines represent time series data from individual animals, dashed line represents average across the respective group, n = 6 animals per group.

To further test the hypothesis that changes in pulsatile lymph movement in distal lymphatics reflect changes in post-tumor lymphatics we measured the rate of probe accumulation and clearance within the inguinal LN during constant intradermal infusion of probe downstream of the tumor. The fluorescent probe exhibited a more rapid arrival and clearance time course in C6 tumor-bearing versus naïve animals (Figure 6), indicating that tumor-induced elevation of pulsatile frequency reflects an increase in lymph movement. This increase in lymph transport distal to the primary tumor was reduced in anti-VEGF-C treated animals. This finding is consistent with previous reports that overexpression of VEGF-C increased lymph flow to SLN [31]. Collectively, our data indicated that the VEGF-C pathway modulates lymphatic function both upstream and downstream of SLNs even when initial lymphatics around primary tumors are not altered.

### VEGF-C Inhibition Prevents Structural Remodeling of Distal Tumor-associated Lymphatic

**Figure 7:**
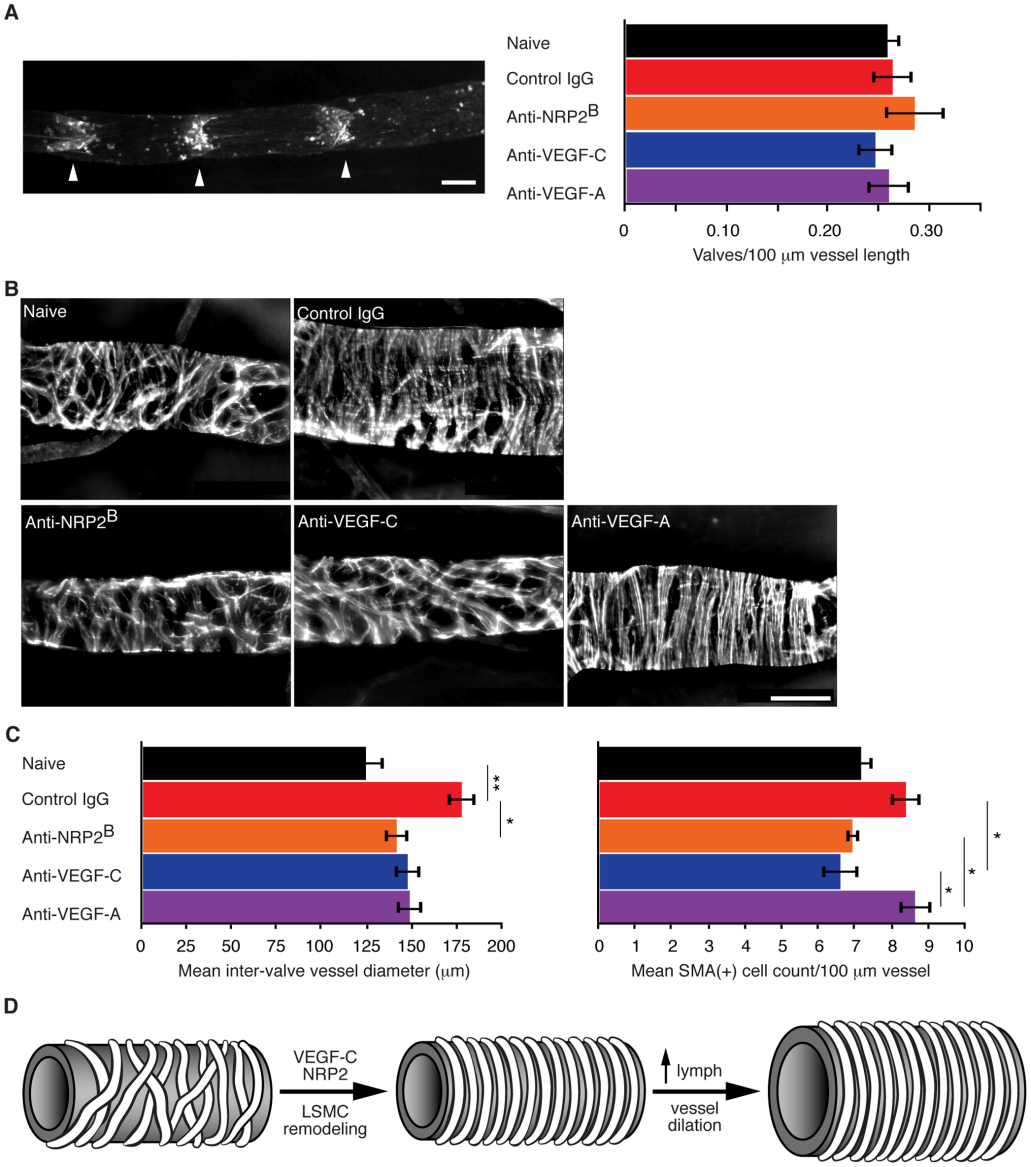
VEGF-C signaling promotes structural remodeling of established lymphatic vessels in metastatic disease model. (A) Representative epi-fluorescence image (left) of an Ing to Ax lymphatic vessel labeled *via* intradermal injection of FITC-Lectin. Right, mean valve density ± SEM per C6 tail xenograft tumor and treatment condition: naïve, 0.259±0.013; control IgG, 0.264±0.020; anti-NRP2^B^, 0.286±0.030; anti-VEGF-C (VC1.12), 0.247±0.018; anti-VEGF-A, 0.260±0.021 valves/100 µm vessel length, n = 5 animals per group. (B) Representative micrographs of αSMA-positive LSMCs along the Ing to Ax vessel. (C) Left, mean Ing to Ax lymph vessel diameter ± SEM measured mid-point between valves: naïve, 125.0±10.5; control IgG, 178.3±8.1; anti-NRP2^B^, 142.1±6.9; anti-VEGF-C (VC1.12), 148.2±7.5; anti-VEGF-A, 149.4±7.6 µm, n = 14, 12, 13, 12, 14 animals per group, respectively. Right, mean LSMC density ± SEM estimated between lymph valves along the Ing to Ax vessel per condition: naïve, 7.20±0.34; control IgG, 8.41±0.43; anti-NRP2^B^, 6.95±0.18; anti-VEGF-C (VC1.12), 6.63±0.51; anti-VEGF-A, 8.71±0.44 LSMC/100 µm vessel, n = 7, 5, 6, 8, 7 animals per group, respectively. (D) Model of tumor-associated structural remodeling of distal lymphatics. Scale bar equals 100 µm in A and B. For indicated treatment conditions, tumor-bearing animals were dosed weekly for three weeks starting two days after xenograft implantation.

During development VEGF-C and NRP2 are required for the formation of normal lymphatics. To test the hypothesis that this pathway is utilized post development to promote structural remodeling of established lymphatic vessels, we characterized the structure of the inguinal to axillary lymphatic vessel across disease and treatment conditions. Collecting lymphatic vessels contain regularly spaced valves that act to prevent backflow of lymph through the vessel, and thus promote unidirectional lymph movement. Valves were stained by intradermal injection of FITC-lectin and visualized within a large field of view using a stereomicroscope to enable quantification of valve distribution across the inguinal to axillary vessel (Figure 7A). Although lymphatic pulsatile frequency was increased in control IgG treated C6 tail xenograft tumor-bearing animals compared to naïve animals, valve density or morphology was not altered compared to naïve animals, nor altered with anti-NRP2^B^, anti-VEGF-C or anti-VEGF-A treatment, suggesting that valve density does not account for the modulation in lymph movement.

Another characteristic feature of collecting lymphatic vessels is their coverage by lymphatic-associated smooth muscle cells (LSMCs), which provide contractility to lymphatic vessels and thus contribute to the propulsion of lymphatic fluid [49], [50]. To quantitatively assess lymphatic vessel morphology and LSMC coverage, inguinal to axillary vessels were stained whole mount using an antibody against alpha-smooth muscle actin (αSMA) then visualized by epifluorescence microscopy (Figure 7B). Strikingly, two morphological features differed between naïve and control IgG treated C6 tumor-bearing animals. First, the mid-lymphangion vessel diameter increased ∼ 40% in tumor-bearing animals over naïve animals (Figure 7C). Second, the orientation of LSMCs along the lymph vessel differed between tumor-bearing and naïve animals. In naïve animals, LSMCs were distributed in an intersecting pattern along the length of the vessel. In tumor-bearing animals, however, LSMC density appeared to increase with cells being aligned perpendicular to the longitudinal axis of the vessel, reminiscent of smooth muscle cell alignment along arteries. Histological analysis utilizing Ki-67 as a marker for cell proliferation did not reveal an obvious increase in lymphatic endothelial cell proliferation (data not shown). Together, these morphometric changes suggest that tumor-associated distal lymphatic vessels undergo structural remodeling to promote increased lymph transport.

If remodeling of LSMCs along lymph vessels drives the observed tumor and VEGF-C-dependent increase in lymph transport, then inhibition of VEGF-C should prevent remodeling. To test this hypothesis, we inhibited the VEGF pathway in C6 tail xenograft tumor-bearing animals then assessed lymphatic vessel morphology. In tumor-bearing animals dosed with anti-NRP2^B^, anti-VEGF-C or anti-VEGF-A antibodies, lymph vessel diameter was reduced to levels comparable to naïve animals (Figure 7C). However, in anti-NRP2^B^ and anti-VEGF-C, but not anti-VEGF-A treated tumor-bearing animals, LSMC orientation resumed the intersecting pattern along the lymph vessel, similar to naïve animals. LSMC morphology in the anti-VEGF-A treated animals remained the same as control IgG treated tumor-bearing animals. Furthermore, the abundance of LSMCs along the vessels was reduced by anti-NRP2^B^ and anti-VEGF-C treatment, whereas anti-VEGF-A had no effect on LSMC density.

Taken together our histological analysis of lymphatic vessel morphology revealed an unexpected pathophysiology of metastatic disease, and a new role for VEGF-C in modulating mature lymphatics. Specifically, during metastatic disease progression, LSMCs reorient along distal tumor-associated lymphatic vessels *via* a VEGF-C and NRP2-dependent pathway (Figure 7D). The diameter of tumor-associated vessels also increase, likely due to increased lymph volume, since anti-VEGF-A prevented vessel enlargement but not LSMC remodeling. By extension, these data imply that the inhibition of LSMC remodeling *via* treatment with anti-VEGF-C and anti-NRP2^B^ contributes to the observed reduction in lymphatic pulsatile frequency in tumor-bearing animals.

### VEGF-C can be Targeted in an Intervention Setting to Normalize Lymph Transport through Distal Tumor-associated Lymph Vessels

**Figure 8:**
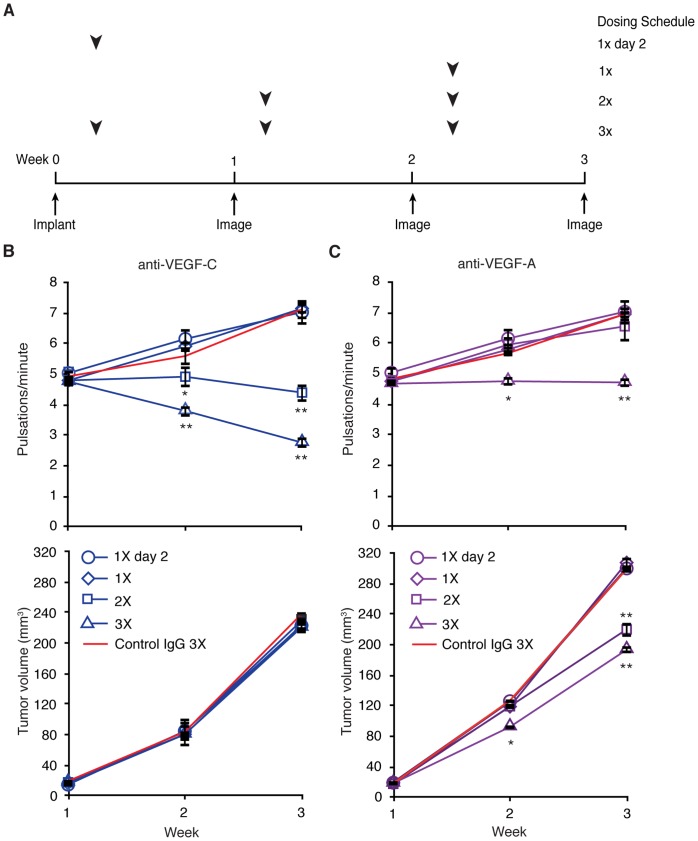
Intervention dosing with anti-VEGF-C antibody mitigates up-regulation of lymph transport. (A) Dosing schedule for intervention study design. Arrowheads denote the timing of dose(s) per schedule relative to C6 tail xenograft tumor implantation and imaging sessions. Imaging occurred at 1 week intervals initiated after tumor implantation, while dosing was offset +2 days relative to tumor implantation and weekly imaging, as indicated. (B) Average lymphatic pulsatile frequency and tumor volume ± SEM measured longitudinally in IgG control and anti-VEGF-C (VC4.5) treated cohorts as a function of dosing scheme, n = 5 animals per condition. (C) Average lymphatic pulsatile frequency and tumor volume ± SEM measured longitudinally in IgG control and anti-VEGF-A treated cohorts as a function of dosing scheme, n = 5 animals per condition. Asterisks indicate comparison of treatment group to IgG control at each time point.

To test if lymphatic remodeling can be inhibited by intervention dosing paradigms, thus potentially useful as a therapeutic target to reduce metastatic spread, we compared lymphatic pulsatile frequency longitudinally across four different dosing schedules in C6 tail xenograft tumor-bearing animals (Figure 8A). Initiating weekly anti-VEGF-C dosing two days after cell implantation (thus 3 doses total over disease course) resulted in incremental reduction of lymphatic pulsatile frequency at weeks 2 and 3 (i.e. after the second and third doses of anti-VEGF-C, respectively, Figure 8B, 3X) compared to animals dosed with control IgG. Furthermore, treatment initiated one week after tumor cell implantation (two doses total) was also able to reduce lymphatic pulsation frequency (Figure 8B, 2X). However, a single dose of anti-VEGF-C either before week 1, or two weeks after tumor cell implantation, did not alter lymphatic pulsation frequency, suggesting that a threshold duration of inhibition and/or dose number is required to reverse tumor-induced modulation of lymph transport.

In contrast to anti-VEGF-C, interventional dosing with anti-VEGF-A did not alter lymph transport (Figure 8C). Initiating treatment two days after cell implantation prevented up-regulation of lymph transport (Figure 8C, 3X), consistent with our previous observation. However, a single dose at day 2 or initiating dosing after week 1 did not reduce pulsatile frequency.

These data suggest that lymphatic functional remodeling could potentially be therapeutically targeted by inhibiting VEGF-C signaling and further support the notion that VEGF-A and VEGF-C act through distinct mechanisms to modulate lymphatic function.

### Therapeutic Inhibition of VEGF-C Decreases Metastatic Spread in an Adjuvant Treatment Model

**Figure 9:**
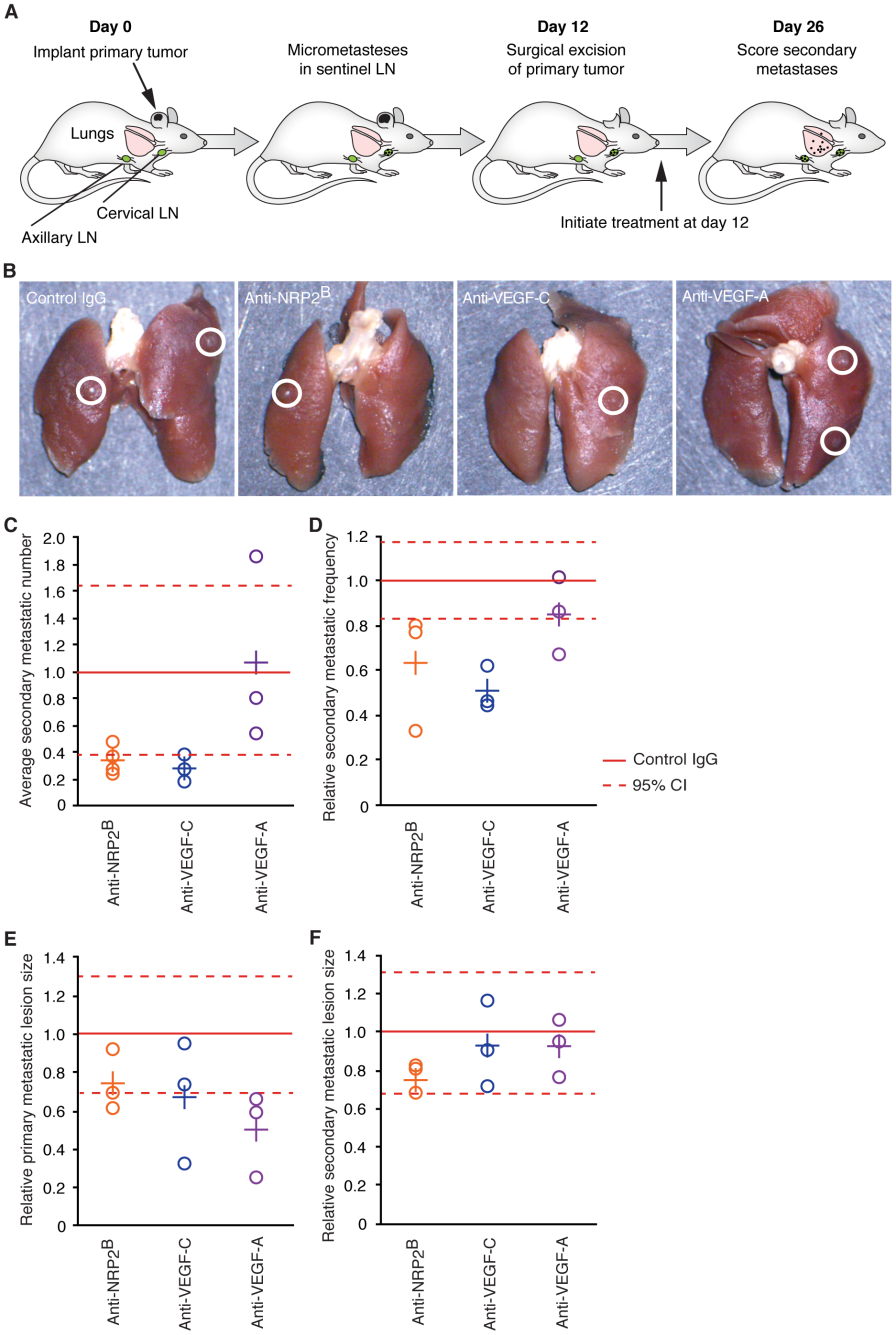
Inhibiting VEGF-C, but not VEGF-A, reduces secondary metastatic spread to the lung in a model of adjuvant therapy. (A) Schematic of the adjuvant therapy model. (B) Representative images of lungs collected 14 days post primary tumor resection, circles indicate metastatic lesions. Animals were dosed weekly with the indicated antibodies starting at day 12, the day of ear resection. Anti-VEGF-C clone VC4.5 was used for two experiments, VC1.12 for the third, in all cases the results were comparable. (C and D) Quantification of the average number of secondary metastatic lesions per animal or the frequency of animals containing at least one secondary metastatic lesion, respectively, plotted per experiment normalized to IgG control (O) and as the average across experiments (+) per treatment condition. (E and F) Relative size of primary and secondary metastatic lesion size, respectively, plotted per experiment normalized to IgG control (O) and as the average across experiments (+) per treatment condition. As plots represent multiple experiments, the mean (–) and 95% confidence intervals (- -) are plotted, instead of SEM, for comparison.

Inhibition of VEGF-C during primary tumor formation has been shown to reduce tumor lymphangiogenesis and SLN metastasis in mouse models [32]–[34], [36]. Furthermore, SLN tumors that expressed VEGF-C have been shown to exhibit an increased propensity to promote distal metastatic dissemination [51]. However, it remains untested if inhibition of VEGF-C, and presumably disease-induced lymphatic remodeling, can prevent disease spread beyond primary metastatic lesions in SLNs–a question of clinical relevance in the adjuvant setting. To address these questions, we sought to develop a preclinical murine model of adjuvant therapy, in which primary xenograft tumors could be established, enabled to ‘seed’ primary metastatic sites such as in the SLNs, then be fully resected well before metastatic cells can be detected in secondary metastatic sites. Implanting C6 xenograft tumors subdermally into the tip of the mouse ear resulted in temporally defined seeding of the SLN by day 12 that preceded detectable seeding of the lung (Figure 9A and Figure S4A), and was coincident with elevated lymph transport through the cervical lymph network (Figure S4B). Therefore, full resection of the ear at day 12 enabled experimental evaluation of adjuvant therapy.

To test if targeting the VEGF-axis could impact secondary spread of tumor cells beyond SLNs, we conducted multiple large cohort experiments in which dosing of anti-NRP2^B^, anti-VEGF-C, or anti-VEGF-A antibodies initiated after primary tumor resection at day 12, then quantified metastases within lungs at day 26. In all replicated experiments, treatment with anti-NRP2^B^ or anti-VEGF-C reduced the average number of metastatic lesions within lungs compared to dosing with control IgG or anti-VEGF-A (Figure 9, B and C). Importantly, anti-NRP2^B^ and anti-VEGF-C treatment also consistently reduced the frequency of lung lesion-positive animals within these treatment groups compared to IgG control and anti-VEGF-A (Figure 9D). This observed decrease in metastatic spread to distant organ does not likely reflect inhibition of primary metastatic growth, as SLN tumor volumes were not consistently decreased with anti-NRP2^B^ or anti-VEGF-C treatment (Figure 9E). Nor does reduced metastatic spread likely reflect decreased growth of secondary metastatic lesions within the lungs, if they occur, as relative secondary lesion sizes did not differ between treatment conditions (Figure 9F). Thus, the reduction of metastatic dissemination beyond SLNs via inhibition of NRP2 or VEGF-C likely results from a decrease in metastatic cell spread from primary (i.e. the SLN) to secondary metastatic sites.

## Discussion

Lymphogenous spread of tumor cells to sentinel lymph nodes is a signature feature of metastatic disease associated with many solid tumors. While a mechanistic understanding of how pro-lymphangiogenesis signaling promotes cell transport to SLNs has been proposed [23], [24], [27], [28], [30], [31], [34]–[36], little is known regarding the behaviors and contribution of distal lymphatic vessels in the further spread of metastatic tumor cells after they exit SLNs; the latter representing a clinically important question as the majority of patients are diagnosed with existing SLN metastases. Thus, inhibition of further tumor cell dissemination would require targeting pathways relevant to post SLN spread.

Non-invasive imaging has been used to study the relationship between peritumoral lymph transport and primary metastases in SLNs [30], [31]. Lymph transport is observed to increase coincident with metastatic spread to SLNs, though full involvement of the SLN or its afferent vessel by metastatic tumor growth can impinge upon lymph movement [37]. We utilized longitudinal imaging of lymph transport to determine if and when distal lymphatic vessels respond to metastatic disease progression, and if this process could be reversed through interventional treatment. Our results suggest that established distal tumor-associated lymphatic vessels undergo VEGF-C-dependent structural and functional remodeling during disease progression. The observed increase in transport of lymph could presumably correspond to an increase in the rate of tumor cell transport through lymphatic vessels, and thus promote metastatic dissemination. It is also possible that other aspects of VEGF-C-dependent lymphatic remodeling could promote metastatic disease. The relative contributions of VEGF-C-dependent modulation of primary tumor-associated lymphatics, immediate collecting lymphatics or SLN lymphatics to increased lymph transport through distal lymphatics remains to be determined. It is also possible that the observed changes in distal lymphatics represent a secondary consequence of VEGF-C-dependent changes in the upstream lymph-draining network. Independent of mechanism, the ability to therapeutically intervene utilizing function blocking antibodies to VEGF-C or NRP2 and to reduce metastatic spread beyond SLNs provides novel experimental evidence that targeting pro-lymphangiogenic pathways could be useful in adjuvant therapy of solid tumors.

In contrast to VEGF-C, antagonism of VEGF-A in the intervention setting did not reduce tumor-associated lymphatic remodeling nor metastatic spread in our model of adjuvant therapy, though disease associated vessel enlargement was reduced, likely due to decreased mechanical induction by lymph pressure [48], [52], [53]. This result suggests that VEGF-A inhibition does not directly impact the biological processes promoting metastatic spread, one of which potentially being lymphatic remodeling. Therefore, these data could help explain recent clinical trials in which anti-VEGF-A based therapeutics did not provide benefit in preventing metastasis [54].

Furthermore, the clinical path forward for developing therapeutics specifically targeting metastatic disease remains challenging due to technical limitations beyond understanding the biology of metastasis. Current clinical endpoints to assess metastasis, for example, rely on measuring progression free survival. As metastatic spread occurs stochastically over years, clinical trials must be designed specifically to measure sporadic disease occurrence on the timescale of years and in the context of multiple and evolving treatment paradigms. The imaging approach outlined in this study, however, could expedite clinical development of anti-metastatic therapeutics that impact disease-associated lymphatic remodeling. Our results suggest that direct drug effect could be measured within patients longitudinally by assessing lymphatic pathophysiology. Several clinically relevant imaging modalities, including near-infrared fluorescence imaging, can be used to visualize lymphatic and lymph movement [30], [55]–[57]. Development of these methods to image lymph movement in relevant metastatic patient populations would provide a pharmacodynamic readout of drug activity, which could enable or even expedite dose selection for long-term efficacy studies.

## Supporting Information

Figure S1 **Physiological modulation of pulsatile lymph movement.** (A) To test which physiological conditions may alter the pulsatile movement of lymph through the inguinal to axillary lymphatic vessel, the frequency of lymphatic pulsation was compared among the following groups of mice: 2-month old adult BALB/c females and males, 15-day old BALB/c females, 9-month old BALB/c females, 2-month old C57BL/6 albino females. Comparison was also carried out between different degrees of anesthesia or body temperatures. (B) Heart and respiratory rate measured by a pulseoximeter during image acquisition for experiments described in A. Bar graphs represent mean ± SEM, n = 5 animals per group.
(TIF)

Figure S2 **Characterization of anti-VEGF-C (VC1.12) antibody.** (**A**) Dose response curve of anti-VEGF-C (VC1.12) or VEGFR3ecd-Fc inhibition of VEGF-C binding to immobilized VEGFR3ecd, ecd = extracellular domain. (**B**) Affinity of anti-VEGF-C (VC1.12) for human, h, or murine, m, VEGF-C as determined by BIAcore Surface Plasmon Resonance measurement. (**C**) Anti-VEGF-C (VC1.12) blocks VEGF-C induced cell migration. Quantification of Lymphatic Endothelial Cell (LEC) migration in response to 200 ng/ml VEGF-C in the presence or absence of anti-VEGF-C (VC1.12) at 50 µg/ml, n = 6 replicates for each condition. “Cell counts” on the x-axis refers to migrated cell counts. (**D**) Anti-VEGF-C (VC1.12) blocks VEGF-C induced proliferation. Quantification of LEC proliferation in response to 200 ng/ml VEGF-C in the presence or absence of anti-VEGF-C (VC1.12) at 50 µg/ml, n = 6 replicates for each condition. Error bars represent SEM in C and D. **Methods; Antibody generation.** Anti-VEGF-C was isolated from antibody phage libraries constructed as previously described (Lee, C. V. et al, J. Mol. Biol. 340, p1073–1093, 2004) by selection against recombinant VEGF-homology domain (VHD) of human VEGF-C. The clone (VC1) that exhibited competitive binding against VEGFR3 was further affinity improved to generate clone VC1.12 by stringent selection from phage displayed libraries of VC1 variants randomized in several CDR loops as previously described (Lee, C. V. et al. Blood, 108, p3103–3111, 2006). **Competitive binding assay** Serial dilutions of anti-VEGF-C or soluble VEGFR3 extracellular domain (ecd) fused to Fc were incubated with a fixed concentration of biotinylated human VEGF-C in solution for one hour. The mixtures were then added to ELISA wells coated with immobilized VEGFR3ecd, which captured unbound biotinylated VEGF-C (i.e. free VEGF-C that was not bound by the anti-VEGF-C antibody or VEGFR3-ecd-Fc in solution). The captured unbound VEGF-C was quantified by incubation with avidin conjugated to horseradish peroxidase (HRP) then detected by an HRP colorimetric substrate. **Affinity determination with BIAcore3000** BIAcore SPR measurement was performed with human or murine VEGF-C immobilized on sensor chips *via* amine coupling linkage at low density (<30 RU) and serial dilutions of IgG were injected as analyte in HEPES buffer. The kinetic parameters were derived from global-fitting of the binding curves using bivalent binding model. **Cell lines**. HMVEC-dLyAd - Human Dermal Lymphatic Microvascular Endothelial Cells (LECs) and HUVECS were purchased from Lonza and cultured in EGM-2 medium (Lonza). **Cell migration assay.** Migration assays were performed using a modified Boyden chamber with 8 µM pore size Falcon 24-multiwell insert system (BD Biosciences). The plates were coated with 5 µg/ml Fibronectin (Invitrogen) for 2 hours at 37°C. Cells in 100 µl assay medium (0.1% BSA, EGM-2) with or without antibodies were added to the upper chamber. Chemoattractant (VEGF-C in this case) was added to the lower chamber in 500 µl assay medium, and cells were incubated at 37°C for 18 hours. Cells on the upper membrane were removed with a sponge swab and cells on the lower surface were fixed in 70% ethanol and stained with Sytox green (Molecular Probes). Images were taken of the entire lower surface of the well, and number of migrated cells counted (6 wells per condition). Isotype control anti-ragweed was used as the control antibody. **Cell proliferation Assays.** A 96-well black-clear bottom plate (VWR) was coated with 5 µg/ml Fibronectin (Invitrogen) at 37°C for 2 hours. LECs were harvested and resuspended in assay medium (0.1% BSA, EGM-2) at 3000 cells per 100 µl and added to wells. Cells were incubated at 37°C for 16 hours. BrdU labeling solution (Cell Proliferation ELISA kit; Roche) was added and the cells were incubated for an additional 24 hours at 37°C. BrdU incorporation was determined by chemiluminescence immunoassay (6 wells per condition). Isotype control anti-ragweed was used as the control antibody.
(TIF)

Figure S3 **Quantification of primary tumor lymphatics.** (**A**) Representative tumor slice for each indicated condition stained with DAPI in blue, anti-LYVE-1 in green, and anti-F4/80 in red. Scale bar = 1 mm. (**B**) Close-up views of boxed regions in panel A. White lines outline the boundaries between tumor and stroma. Objects that are double positive for anti-LYVE-1 and anti-F4/80 (appear yellow in the close-up panels) were removed from the lymphatic vessel quantification, as described below. Scale bar = 200 µm. (**C**) and (**D**) Quantification of lymphatic vessel density and lymphatic vessel diameter, respectively, plotted as mean ± SEM. Numbers of mice are: 6, 6, 5 and 7 for control IgG, anti-NRP2^B^, anti-VEGF-C (VC1.12) and anti-VEGF-A, respectively. In comparison to control IgG, by one-way ANOVA with *posthoc* Tukey-Kramer HSD test, lymphatic vessel density and diameter was not significantly different in anti-NRP2^B^, anti-VEGF-C (VC1.12) or anti-VEGF-A treated groups, p>0.1. In this study, we did not evaluate the impact of tumors on peritumoral lymphatic vessel diameter, which has been reported to increase (Hagendoorn et al 2006, Hoshida et al 2006, Karnezis et al 2012). What was measured in this experiment represents treatment effect only. **Methods Immunohistochemistry.** Mice were perfused with 4% PFA and tumors harvested. Tumors were fixed overnight in 4% PFA at 4°C, then changed to 70% EtOH overnight. Tumors were then embedded in paraffin and sectioned. Sections were antigen retrieved for 20 minutes at 99°C in target retrieval solution (Dako), followed by 20 minutes at room temperature. Sections were blocked with 0.1% BSA, 10% donkey serum in PBS for 1 hour at room temperature, then incubated with anti-LYVE-1 (1∶200; R&D systems) and anti-F4/80 (1∶100, Serotec) overnight at 4°C. Samples were stained with Alexa-594 or 488 conjugated secondary antibodies (1∶500; Molecular Probes) for 1 hour at room temperature and counterstained with DAPI. **Imaging & Image Analysis.** Samples were imaged at 10x with a Leica Ariol SL50 Slide Scanner at 10x. Analysis was performed using custom-written MATLAB code. Briefly, tissue and tumor-stromal boundaries were defined based on DAPI density followed by user-guided correction in several cases. Since anti-LYVE-1 is known to also label macrophages, macrophage objects were segmented by applying an image-specific global threshold to the F4/80 image, and then subtracted from the LYVE-1 positive objects. In addition, we observed autofluorescence of red blood cells (RBCs) in all channels, therefore, we acquired a background fluorescence image in the cyan channel that represents RBC autofluorescence, and subtracted it from the anti-LYVE-1 image. Lymphatic vessel objects were then identified by local thresholding and further selected based on eccentricity (aspect ratio >4), tortuosity (solidity <0.55), and size (area >584 µm^∧^2) in order to exclude single round cells. Lymphatic vascular density was calculated as total vessel object area over tissue area (either intratumoral or peritumoral area).
(TIF)

Figure S4 **Characterization of metastatic disease progression in a preclinical adjuvant model of therapy.** (**A**) Temporal resolution of metastatic progression. To establish the timing of tumor cell arrival within SLN versus distant organs (lungs), C6-LacZ xenograft tumors were implanted into the tip of the ear then detected within harvested SLN and lungs at various time points post-implantation. Specifically, cohort of 5 animals were sacrificed on days 0, 1, 2, 3, 6, 9, 12, 15, 18, 24, and 30 to collect the lungs and superior cervical nodes, which represent the SLN relative to the ear. The presence of tumor cells in collected tissue was detected based on LacZ activity in lysates. Specifically, SLNs or lungs were homogenized with M-PER reagent, then samples were incubated with β-Gal Assay Reagent followed by Stop solution (β-Gal Assay Kit, Pierce Biotechnology Cat# 75707) and the product measured by absorbance at 405nm using a plate reading spectrophotometer (SpectraMax M5, Molecular Devices). The lower limit of cell detection for this method was determined *via* serial dilutions of C6-LacZ cells spiked into control tissue samples, ∼10 C6-LacZ cells could be detected within a tissue lysate (data not shown). Based on this approach, the timing of SLN seeding is between day 3 and 12 post implantation, as 100% of the harvested SLN from the day 12 cohort contained LacZ-positive cells. LacZ-positive cells were not detected in lungs until day 18 post implantation. At day 30, 100% of the harvested lungs contained LacZ activity. (**B**) Increased lymph transport in distal lymphatic vasculature associated with ear xenograft tumors. Due to the anatomy of the cervical lymph network, collecting vessels could not be visualized; therefore we assessed lymph transport within this network *via* visualization of probe accumulation within the cervical lymph node, which represents the SLN, during constant subcutaneous infusion (3.4 µL/min for duration of imaging) of probe at the base of the ear. Quantification of fluorescence intensities within a region of interest containing the cervical LN reveals that the probe accumulates within the cervical LN more rapidly in ear xenograft tumor-bearing animals compared to naïve animals. Thin lines represent time series data from individual animals, dashed line represents average across the respective group, n = 6 control and 10 tumor-bearing animals.
(TIF)
