# Supplementary figures and images for: Inhibition of VEGF-C Modulates Distal Lymphatic Remodeling and Secondary Metastasis
Source: PLoS One. 2013 Jul 16;8(7):e68755. doi: 10.1371/journal.pone.0068755 (PMC3712991; doi:10.1371/journal.pone.0068755)

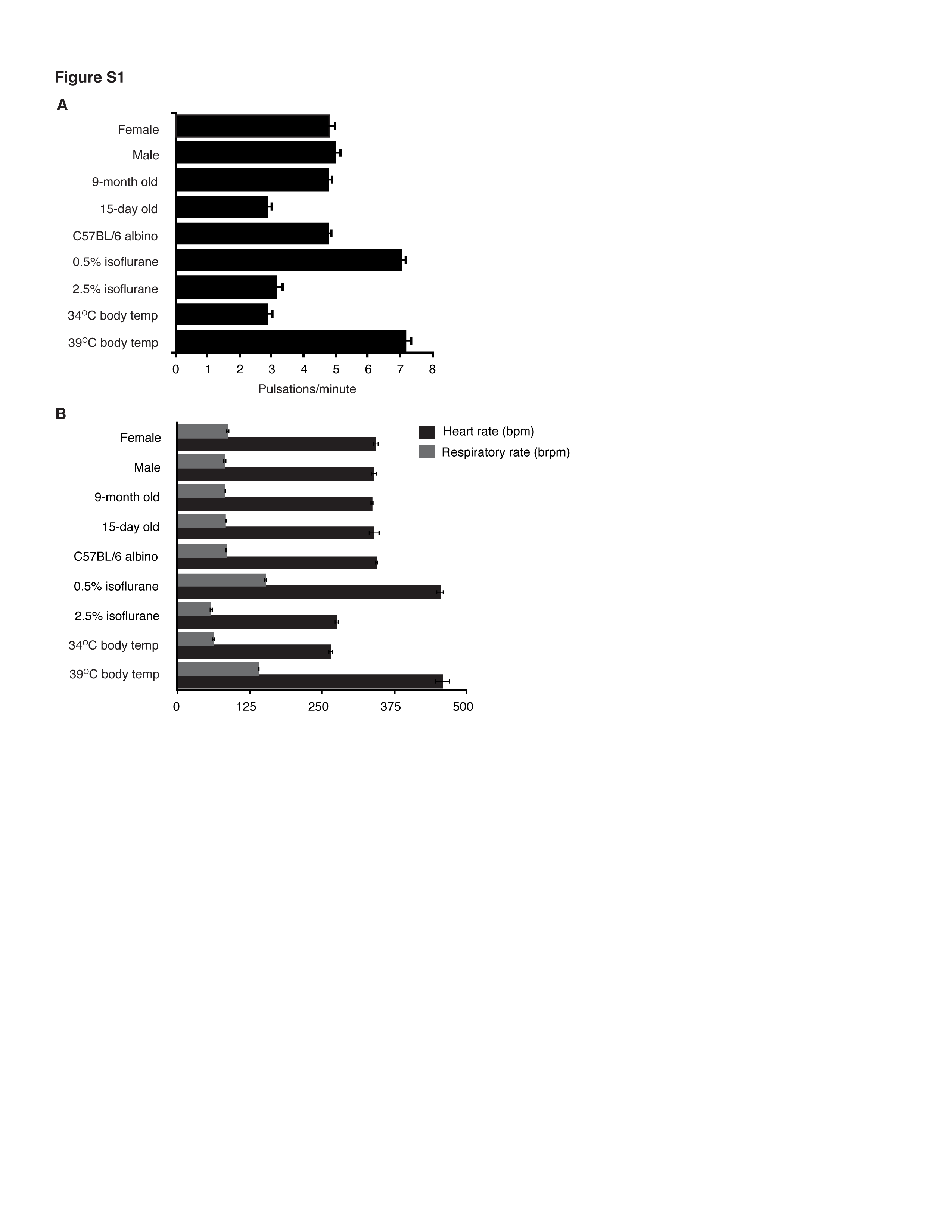

Supplement: Figure S1 — Physiological modulation of pulsatile lymph movement. (A) To test which physiological conditions may alter the pulsatile movement of lymph through the inguinal to axillary lymphatic vessel, the frequency of lymphatic pulsation was compared among the following groups of mice: 2-month old adult BALB/c females and males, 15-day old BALB/c females, 9-month old BALB/c females, 2-month old C57BL/6 albino females. Comparison was also carried out between different degrees of anesthesia or body temperatures. (B) Heart and respiratory rate measured by a pulseoximeter during image acquisition for experiments described in A. Bar graphs represent mean ± SEM, n = 5 animals per group. (TIF) [file pone.0068755.s001.tif]

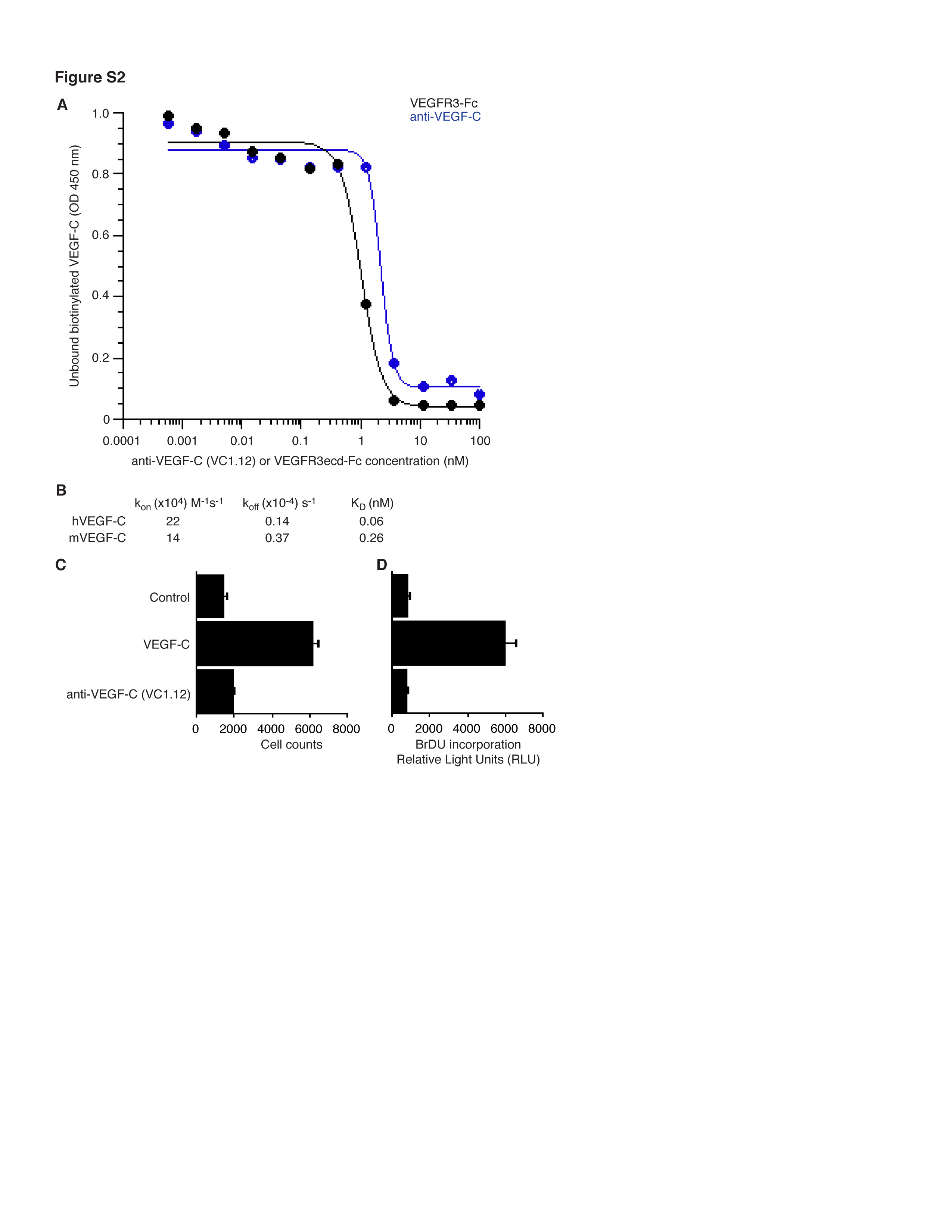

Supplement: Figure S2 — Characterization of anti-VEGF-C (VC1.12) antibody. (A) Dose response curve of anti-VEGF-C (VC1.12) or VEGFR3ecd-Fc inhibition of VEGF-C binding to immobilized VEGFR3ecd, ecd = extracellular domain. (B) Affinity of anti-VEGF-C (VC1.12) for human, h, or murine, m, VEGF-C as determined by BIAcore Surface Plasmon Resonance measurement. (C) Anti-VEGF-C (VC1.12) blocks VEGF-C induced cell migration. Quantification of Lymphatic Endothelial Cell (LEC) migration in response to 200 ng/ml VEGF-C in the presence or absence of anti-VEGF-C (VC1.12) at 50 µg/ml, n = 6 replicates for each condition. “Cell counts” on the x-axis refers to migrated cell counts. (D) Anti-VEGF-C (VC1.12) blocks VEGF-C induced proliferation. Quantification of LEC proliferation in response to 200 ng/ml VEGF-C in the presence or absence of anti-VEGF-C (VC1.12) at 50 µg/ml, n = 6 replicates for each condition. Error bars represent SEM in C and D. Methods; Antibody generation. Anti-VEGF-C was isolated from antibody phage libraries constructed as previously described (Lee, C. V. et al, J. Mol. Biol. 340, p1073–1093, 2004) by selection against recombinant VEGF-homology domain (VHD) of human VEGF-C. The clone (VC1) that exhibited competitive binding against VEGFR3 was further affinity improved to generate clone VC1.12 by stringent selection from phage displayed libraries of VC1 variants randomized in several CDR loops as previously described (Lee, C. V. et al. Blood, 108, p3103–3111, 2006). Competitive binding assay Serial dilutions of anti-VEGF-C or soluble VEGFR3 extracellular domain (ecd) fused to Fc were incubated with a fixed concentration of biotinylated human VEGF-C in solution for one hour. The mixtures were then added to ELISA wells coated with immobilized VEGFR3ecd, which captured unbound biotinylated VEGF-C (i.e. free VEGF-C that was not bound by the anti-VEGF-C antibody or VEGFR3-ecd-Fc in solution). The captured unbound VEGF-C was quantified by incubation with avidin conjugated to horseradish perox [file pone.0068755.s002.tif]

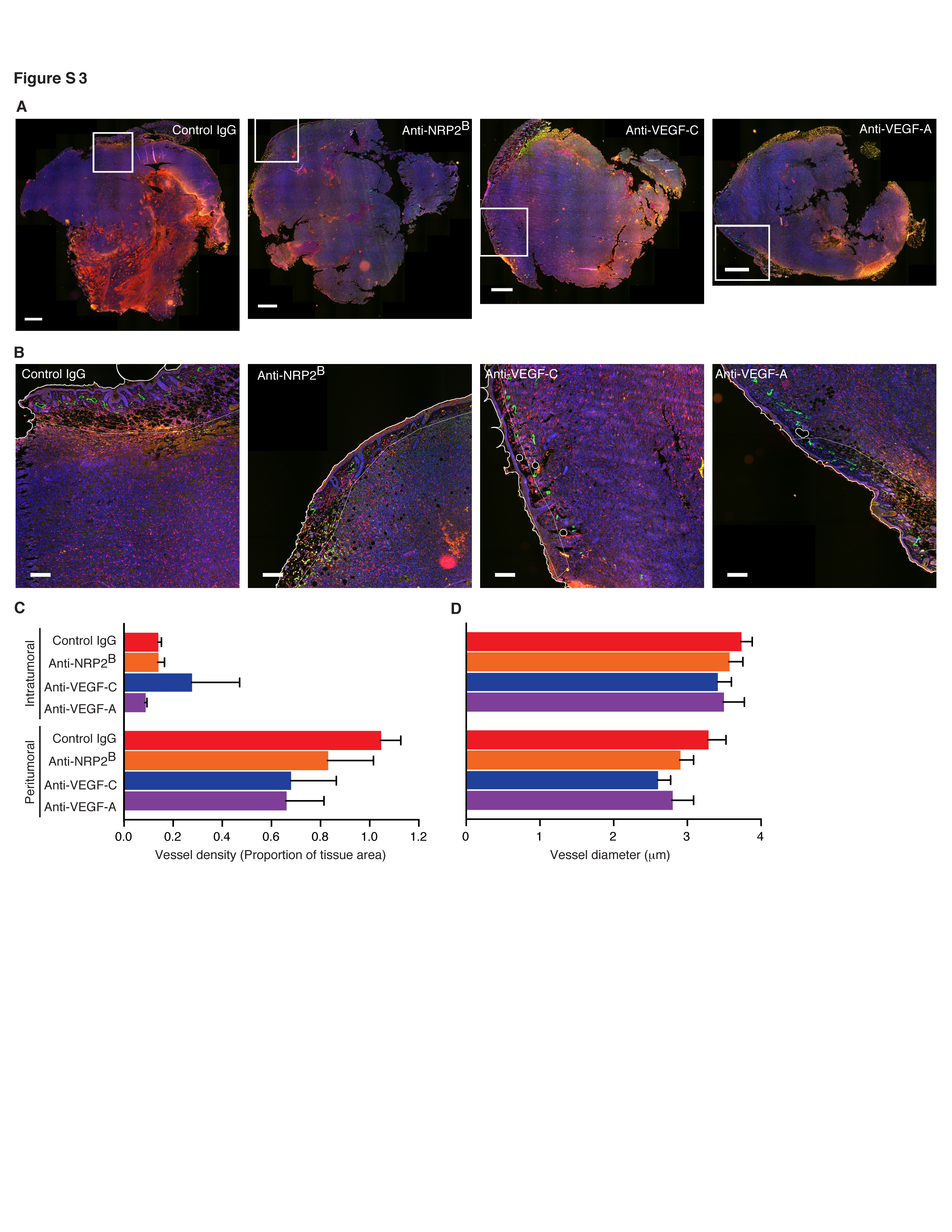

Supplement: Figure S3 — Quantification of primary tumor lymphatics. (A) Representative tumor slice for each indicated condition stained with DAPI in blue, anti-LYVE-1 in green, and anti-F4/80 in red. Scale bar = 1 mm. (B) Close-up views of boxed regions in panel A. White lines outline the boundaries between tumor and stroma. Objects that are double positive for anti-LYVE-1 and anti-F4/80 (appear yellow in the close-up panels) were removed from the lymphatic vessel quantification, as described below. Scale bar = 200 µm. (C) and (D) Quantification of lymphatic vessel density and lymphatic vessel diameter, respectively, plotted as mean ± SEM. Numbers of mice are: 6, 6, 5 and 7 for control IgG, anti-NRP2B, anti-VEGF-C (VC1.12) and anti-VEGF-A, respectively. In comparison to control IgG, by one-way ANOVA with posthoc Tukey-Kramer HSD test, lymphatic vessel density and diameter was not significantly different in anti-NRP2B, anti-VEGF-C (VC1.12) or anti-VEGF-A treated groups, p>0.1. In this study, we did not evaluate the impact of tumors on peritumoral lymphatic vessel diameter, which has been reported to increase (Hagendoorn et al 2006, Hoshida et al 2006, Karnezis et al 2012). What was measured in this experiment represents treatment effect only. Methods Immunohistochemistry. Mice were perfused with 4% PFA and tumors harvested. Tumors were fixed overnight in 4% PFA at 4°C, then changed to 70% EtOH overnight. Tumors were then embedded in paraffin and sectioned. Sections were antigen retrieved for 20 minutes at 99°C in target retrieval solution (Dako), followed by 20 minutes at room temperature. Sections were blocked with 0.1% BSA, 10% donkey serum in PBS for 1 hour at room temperature, then incubated with anti-LYVE-1 (1∶200; R&D systems) and anti-F4/80 (1∶100, Serotec) overnight at 4°C. Samples were stained with Alexa-594 or 488 conjugated secondary antibodies (1∶500; Molecular Probes) for 1 hour at room temperature and counterstained with DAPI. Imaging & Image Analysis. Samples were imaged at 1 [file pone.0068755.s003.tif]

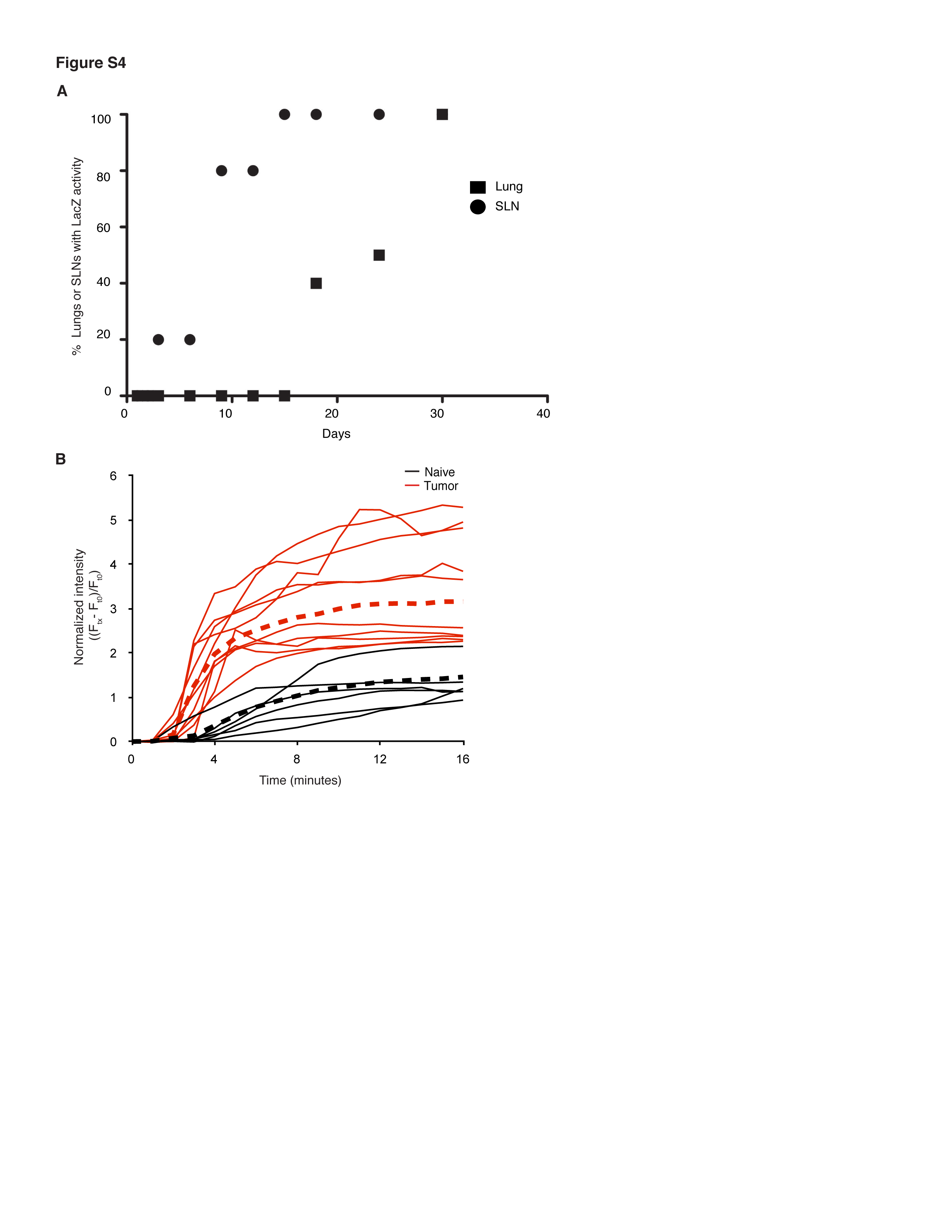

Supplement: Figure S4 — Characterization of metastatic disease progression in a preclinical adjuvant model of therapy. (A) Temporal resolution of metastatic progression. To establish the timing of tumor cell arrival within SLN versus distant organs (lungs), C6-LacZ xenograft tumors were implanted into the tip of the ear then detected within harvested SLN and lungs at various time points post-implantation. Specifically, cohort of 5 animals were sacrificed on days 0, 1, 2, 3, 6, 9, 12, 15, 18, 24, and 30 to collect the lungs and superior cervical nodes, which represent the SLN relative to the ear. The presence of tumor cells in collected tissue was detected based on LacZ activity in lysates. Specifically, SLNs or lungs were homogenized with M-PER reagent, then samples were incubated with β-Gal Assay Reagent followed by Stop solution (β-Gal Assay Kit, Pierce Biotechnology Cat# 75707) and the product measured by absorbance at 405nm using a plate reading spectrophotometer (SpectraMax M5, Molecular Devices). The lower limit of cell detection for this method was determined via serial dilutions of C6-LacZ cells spiked into control tissue samples, ∼10 C6-LacZ cells could be detected within a tissue lysate (data not shown). Based on this approach, the timing of SLN seeding is between day 3 and 12 post implantation, as 100% of the harvested SLN from the day 12 cohort contained LacZ-positive cells. LacZ-positive cells were not detected in lungs until day 18 post implantation. At day 30, 100% of the harvested lungs contained LacZ activity. (B) Increased lymph transport in distal lymphatic vasculature associated with ear xenograft tumors. Due to the anatomy of the cervical lymph network, collecting vessels could not be visualized; therefore we assessed lymph transport within this network via visualization of probe accumulation within the cervical lymph node, which represents the SLN, during constant subcutaneous infusion (3.4 µL/min for duration of imaging) of probe at the base of the ear. Quantificatio [file pone.0068755.s004.tif]
